# Influence of Pre‐Vaccination HPV Status on Vaccine Effectiveness Among Chinese Women: A Multicenter Cross‐Sectional Study

**DOI:** 10.1002/cnr2.70294

**Published:** 2025-08-28

**Authors:** Lucia Li, Haiyue Wu, Yibo Chen, Zhanjun Shen, Kun Fu, Minghui Qiu, Yingzhen Liu, Yufei Shen, Yingnan Lu, Xinxin Wen, Siyu Yang, Kehan Zou, Hui Zhang, Yangzong Gesang, Haojie Huang, Chao Zhao, Pengming Sun, Lisha Wu, Yu Zhang

**Affiliations:** ^1^ Department of Gynecology Xiangya Hospital, Central South University Changsha China; ^2^ Gynecological Oncology Research and Engineering Center of Hunan Province Changsha China; ^3^ National Clinical Research Center for Geriatric Disorders, Xiangya Hospital, Central South University Changsha China; ^4^ Department of Obstetrics & Gynecology Changsha Hospital for Maternal & Child Health Care, Affiliated to Hunan Normal University Changsha China; ^5^ Department of Gynecology Liling Maternal and Child Health Care Hospital Zhuzhou Hunan China; ^6^ Department of Obstetrics and Gynecology Peking University People's Hospital Beijing China; ^7^ Laboratory of Gynecologic Oncology, Department of Gynecology College of Clinical Medicine for Obstetrics & Gynecology and Pediatrics, Fujian Maternity and Child Health Hospital, Fujian Medical University Fuzhou Fujian China; ^8^ Institute of Medical Sciences, Xiangya Hospital, Central South University Changsha China

**Keywords:** Chinese women, human papillomavirus, protective effect, vaccines

## Abstract

**Background and Aims:**

Human papillomavirus (HPV) vaccines have been available in China for only 8 years, and routine HPV testing is not recommended prior to vaccination. Therefore, evaluation of HPV vaccine effectiveness and the impact of pre‐vaccination HPV infection status on vaccine protective effect in Chinese women is warranted.

**Methods:**

From June 2022 to June 2023, women aged 18 to 50 years without a history of cervical or uterine excision were recruited from three medical institutions. Baseline characteristics were compared between vaccinated and unvaccinated participants, with inverse probability treatment weighting (IPTW) applied to adjust for confounding factors. HPV infection rates and vaccine effectiveness (VE) were calculated. Additionally, a sub‐group analysis was conducted among vaccinated women to explore the impact of pre‐vaccination HPV infection status.

**Results:**

After adjusting for group differences, the vaccine effectiveness against new HPV16/18 infections was 76.1% (95% CI: 58.7%–86.2%) among 2285 participants. Older age and possession of a master's degree or higher were identified as protective factors, whereas increased parity and exclusive use of oral contraceptives were determined to be risk factors for HPV16/18 infection. Women with unknown pre‐vaccination HPV status exhibited significantly higher post‐vaccination rates of high‐risk HPV infections (RR 4.278, 95% CI: 2.537–7.215) compared to those who were HPV‐negative prior to vaccination. However, no significant difference in new high‐risk HPV infection rates was observed between pre‐vaccination HPV‐negative and HPV‐positive women.

**Conclusion:**

In addition to HPV vaccination, factors such as age, parity, exclusive use of oral contraceptives, and higher education attainment were independently associated with HPV16/18 infection rates. Pre‐vaccination HPV infection status did not significantly influence the protective efficacy of the HPV vaccine against previously unencountered HPV types.

## 1 Background

Cervical cancer ranks as the fourth most prevalent cancer among women worldwide, posing a significant health threat. In 2020, the age‐standardized incidence rate was approximately 13.3 per 100 000 women, with a mortality rate of about 7.2 per 100 000 women [1].

The primary etiological factor for cervical cancer is persistent infection with Human papillomavirus (HPV), which is common among sexually active women, particularly between the ages of 16 and 25, and can occur even after cervical conization [2, 3]. It is estimated that approximately 70% of women will acquire HPV at some point during their lifetime [4]. Currently, over 220 types of HPV have been identified, with 12 recognized as high‐risk or carcinogenic (hr‐HPV): HPV 16, 18, 31, 33, 35, 39, 45, 51, 52, 56, 58, and 59, along with types 66 and 68, which are considered potentially carcinogenic [5]. These types have been approved by the Food and Drug Administration (FDA) for inclusion in HPV testing during cervical cancer screening [5, 6]. Notably, HPV types 16 and 18 are responsible for approximately 70% of invasive cervical cancer cases [7, 8]. Cervical cancer screening based on HPV and cervical cytology tests such as ThinPrep Cytology Test (TCT) has been proven to be effective in reducing cervical cancer mortality; additionally, the World Health Organization's (WHO) cervical cancer elimination plan further emphasizes the administration of HPV vaccines [9, 10].

Prophylactic HPV vaccines have been developed to prevent HPV infection and HPV‐related diseases [11, 12]. Currently available HPV vaccines consist of bivalent vaccines targeting HPV types 16 and 18, quadrivalent vaccines targeting HPV types 16, 18, 6, and 11, and 9‐valent vaccines targeting HPV types 16, 18, 6, 11, 31, 33, 45, 52, and 58 infections [13, 14]. Global clinical trials have consistently demonstrated the effectiveness of HPV vaccines: the Costa Rica vaccine trial revealed that the bivalent vaccine had a cumulative effectiveness of 97.4% against HPV 16/18‐related cervical intraepithelial neoplasia (CIN) grade 2 or higher, and the FUTURE II study confirmed that the quadrivalent HPV vaccine also provided long‐lasting protection against HPV 16/18‐related cervical lesions over a 14‐year follow‐up period [15, 16].

The implementation of national HPV vaccination programs has effectively reduced the prevalence of HPV infections, thereby reducing cervical cancer incidence [17, 18]. However, studies have shown that hr‐HPV infections and cervical cancer can still occur after vaccination [19], highlighting the importance of analyzing the factors that influence vaccine protective effectiveness and exploring the characteristics of post‐vaccination HPV infections. Although the Advisory Committee on Immunization Practices (ACIP) in the United States does not recommend routine HPV or TCT testing prior to HPV vaccination, the protective effect of HPV vaccines in women with pre‐existing or unknown HPV infection status remains controversial [20, 21, 22].

In China, the most prevalent HPV types are 16, 52, 58, 18, and 33 [23, 24, 25]. The HPV vaccine has been officially available in China for only 8 years, with the 9‐valent formulation available for just 6 years [26]. Clinical trials have confirmed the protective effects of the bivalent vaccine (Cecolin) against HPV16/18‐related high‐grade lesions of the lower reproductive tract [27]. However, given the relatively short period of vaccine availability in China, further research on HPV vaccine effectiveness in the Chinese population is warranted.

Our study aims to evaluate HPV vaccine effectiveness in Chinese women and determine how factors, particularly pre‐vaccination HPV infection status, influence vaccine protective effect. These findings will provide valuable data for healthcare providers and women who may benefit from HPV vaccination.

## 2 Method

### 2.1 Study Population

This multicenter cross‐sectional study recruited eligible women from gynecological clinics and health examination centers across three medical institutions of different tiers in China from June 1, 2022 to June 30, 2023. Inclusion criteria were: (1) females aged 18–50 years; (2) no reported history of cervical cancer or hysterectomy. Exclusion criteria were: (1) inability to cooperate with the investigator; (2) missing key information in the questionnaire; (3) duplicate identification numbers.

### 2.2 Study Design

Data collection was conducted using a questionnaire developed by gynecological experts from participating institutions, based on epidemiological knowledge of HPV and cervical cancer, recommended HPV vaccination strategies, and clinical experience [28, 29]. The complete questionnaire content is presented in Table S1.

For participant recruitment, trained researchers distributed informational posters detailing the research purpose, questionnaire content, and completion requirements. Women who voluntarily participated accessed the electronic questionnaire by scanning a QR code. The study protocol was approved by the ethics committee of the participating hospital (ethics approval number: 202103233).

### 2.3 Main Outcome Measures

Participants were categorized into vaccinated (received HPV vaccine) and unvaccinated groups. Demographic characteristics were compared between groups to identify potential factors influencing vaccination uptake. For participants with available HPV test results, we analyzed the prevalence of hr‐HPV infections, specifically examining the HPV types 16/18, types covered by the 9‐valent vaccine, and potential cross‐protection types (HPV types 31/33/45). We also assessed the incidence of newly acquired HPV infections, defined as hr‐HPV positivity after vaccination in participants who tested negative for corresponding HPV types before vaccination. Vaccine effectiveness was calculated, and the risk factors for post‐vaccination HPV 16/18 infection were analyzed.

Given that HPV results were collected via questionnaires, participants reported results in various forms, including high‐risk or low‐risk HPV status without type specification. For type‐specific HPV infection rate calculations, we used as the denominator only those participants with available type‐specific results. For example, in calculating the HPV16/18 infection rate, we excluded cases where only high‐risk HPV status was reported without type specification.

To evaluate protective effects by pre‐vaccination HPV infection status, we stratified the vaccinated group into three subgroups based on their pre‐vaccination HPV testing status: (1) hr‐HPV negative, (2) HPV status unknown, (3) hr‐HPV positive. We then compared post‐vaccination HPV positivity rates and incidence of newly acquired HPV infections among these subgroups.

### 2.4 Quality Control

To ensure data quality, all key questionnaire items were designated as mandatory fields. Quality control measures were implemented after data collection: questionnaires with duplicate identification numbers were excluded, and responses completely unrelated to questions were coded as unknown (UK). For questionnaires containing ambiguous entries or lacking critical information, such as HPV or TCT test results, we verified data against hospital electronic medical records when valid identification numbers were provided. In cases where verification was not possible, responses were coded as UK and excluded from relevant analysis.

### 2.5 Statistical Analysis

The minimum sample size required to evaluate the protective effect of the HPV vaccine was calculated using the formula: *n* = (*u*_(α/2) √2pq + *u*_*β* √(p0q0 + p1q1))^2^/(p1—p0)^2^, where HPV infection rates were derived from the incidence of opportunistic infections reported in previous studies [30]. The calculation indicated that each group required at least 258 participants.

Continuous variables, such as age, were expressed as mean ± standard deviation (mean ± SD), while categorical variables were expressed as percentages. For between‐group comparisons, continuous variables following a normal distribution (or assumed to be normal due to sufficient sample size) were analyzed using the *t* test, and categorical variables were compared using the chi‐square test.

To address differences in baseline characteristics between vaccinated and unvaccinated groups, we employed the inverse probability treatment weighting (IPTW) method based on propensity scores (PS) to eliminate potential impact from confounding factors. A multivariable logistic regression model incorporating factors showing statistically significant between‐group differences was used to calculate PS for each participant. Inverse probability weights were then derived from the estimated propensity scores. Covariate balance between groups was assessed before and after IPTW using the standardized mean difference (SMD), with an SMD < 0.1 indicating minimal between‐group variation. This approach enabled more accurate outcome comparisons between vaccinated and unvaccinated groups.

HPV infection rates between groups were compared using the chi‐square test. Vaccine effectiveness (VE) and its 95% confidence interval (CI) were calculated using the risk ratio (RR) according to the formula: VE = (1—RR) × 100%. To identify factors influencing post‐vaccination HPV infection, we conducted univariate and multivariate logistic regression analysis. Factors demonstrating statistical significance in the univariate analysis were subsequently included in the multivariate analysis. For all chi‐square tests and logistic regression analysis, *p* value < 0.05 was considered statistically significant.

Statistical analysis was performed using SPSS version 26.0 (IBM Corporation, United States), RStudio (Posit, PBC, United States), and R version 4.3.3 (R Foundation for Statistical Computing, Austria).

## 3 Results

### 3.1 Differences in Baseline Characteristics Between Vaccinated and Unvaccinated Groups

**FIGURE 1:**
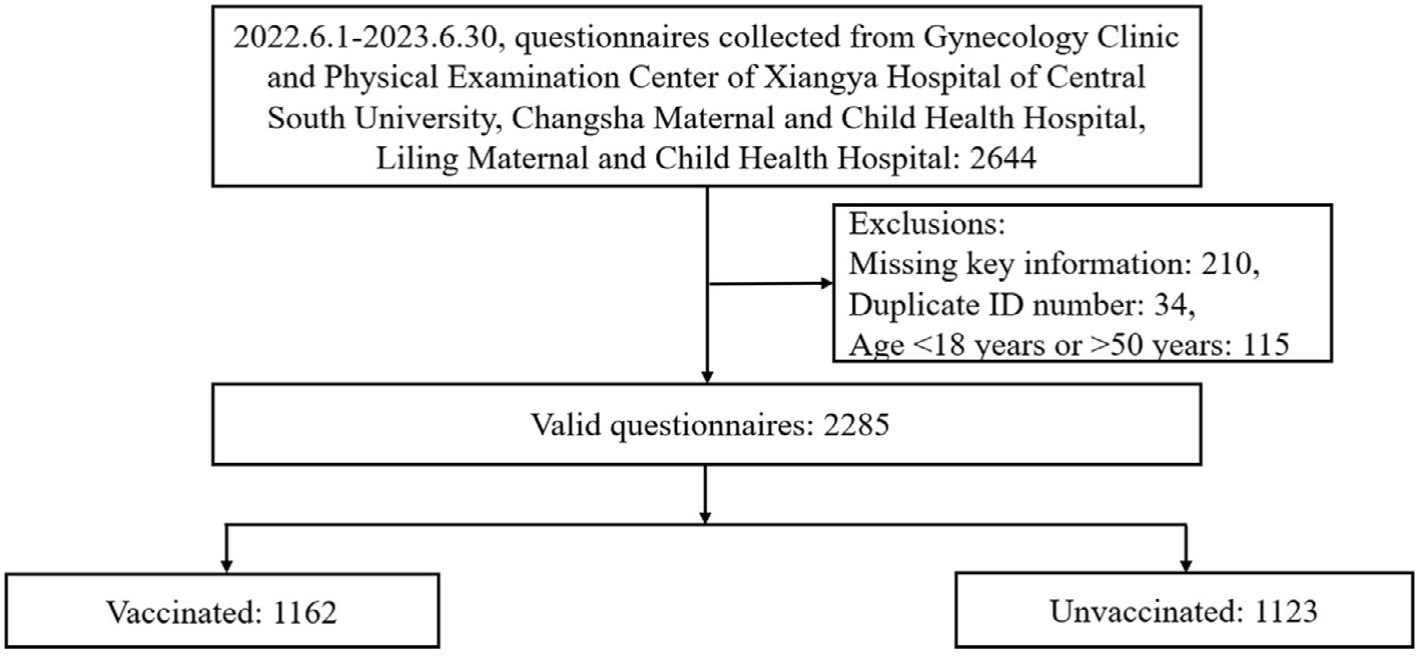
The process of questionnaires collection.

A total of 2644 questionnaires were collected from three hospitals. After excluding questionnaires with missing key information, duplicate identification numbers, and respondents outside the inclusion age range, 2285 valid questionnaires remained. Participants were categorized into two groups based on their HPV vaccination status: the vaccinated group (*N* = 1162) and the unvaccinated group (*N* = 1123) (Figure 1).

As shown in Table 1, the mean age of the vaccinated group was 32.53 ± 6.67 years, which was lower than that of the unvaccinated group (35.25 ± 7.73). The vaccinated group had a higher proportion of women with a bachelor's degree and above (81.7% vs. 70.1%), and a larger percentage of women in the vaccinated group reported a monthly income of 8001–10 000 yuan or above (25.1%) compared to the unvaccinated group (22.3%). Conversely, the proportion of women with a history of pregnancy was lower in the vaccinated group (64.7%) compared to the unvaccinated group (78.1%). Additionally, women in the vaccinated group had fewer average pregnancies (1.50 vs. 1.98) and births (0.92 vs. 1.12). The vast majority of women in both the vaccinated (99.5%) and unvaccinated groups (98.2%) had received other vaccines (including COVID‐19, hepatitis B, influenza, and other participant‐specified vaccines). With respect to contraception, the vaccinated group reported higher condom‐only use compared to the unvaccinated group (51.7% vs. 41.6%).

**TABLE 1:** Basic characteristics of women enrolled.

	Before IPTW				After IPTW			
	Vaccinated *n* = 1162	Unvaccinated *n* = 1123	*p*	SMD	Vaccinated *n* = 2276	Unvaccinated *n* = 2288	*p*	SMD
Age, mean ± SD	32.53 ± 6.67	35.25 ± 7.73	**< 0.001**	**0.376**	33.73 ± 6.81	33.79 ± 7.77	0.839	0.009
Ethnicity, *n* (%)			**0.019**	**0.101**			0.833	0.009
Han	985 (84.8)	1026 (91.4)			2119 (93.1)	2125 (92.9)		
Other	63 (5.4)	97 (8.6)			157 (6.9)	163 (7.1)		
UK	114 (9.8)	0			0	0		
Education, *n* (%)								
Elementary school	2 (0.2)	13 (1.2)	**< 0.001**		9 (0.4)	17 (0.7)	0.859	0.053
Junior high school	63 (5.4)	119 (10.6)			189 (8.3)	177 (7.7)		
High school	148 (12.7)	204 (18.2)			354 (15.6)	360 (15.7)		
Undergraduate	709 (61.0)	633 (56.4)			1328 (58.4)	1354 (59.2)		
Graduate or higher	240 (20.7)	154 (13.7)			395 (17.4)	380 (16.6)		
Monthly income, *n* (%)								
None	107 (9.2)	110 (9.8)	**< 0.001**	**0.213**	238 (10.5)	270 (11.8)	**0.009**	**0.174**
< 2000 CNY	21 (1.8)	44 (3.9)			60 (2.6)	93 (4.1)		
2001–5000 CNY	321 (27.6)	307 (27.3)			758 (33.3)	707 (30.9)		
5001–8000 CNY	282 (24.3)	268 (23.9)			610 (26.8)	619 (27.0)		
8001–10 000 CNY	174 (15.0)	113 (10.1)			355 (15.6)	266 (11.6)		
> 10 000 CNY	117 (10.1)	137 (12.2)			254 (11.2)	334 (14.6)		
UK	140	144			0	0		
Pregnancy history, *n* (%)			**< 0.001**	**0.299**			0.370	0.039
Yes	752 (64.7)	877 (78.1)			672 (29.5)	636 (27.8)		
No	410 (35.3)	246 (21.9)			1603 (70.4)	1653 (72.2)		
Times of pregnancy, mean ± SD	1.50 ± 1.53	1.98 ± 1.61	**< 0.001**	**0.309**	1.75 ± 1.63	1.75 ± 1.57	0.945	0.003
Times of birth, mean ± SD	0.92 ± 0.84	1.12 ± 0.85	**< 0.001**	**0.229**	1.03 ± 0.83	1.03 ± 0.86	0.957	0.002
Smoking, *n* (%)			**0.011**	**0.112**			0.930	0.004
Yes	26 (2.6)	53 (4.7)			2193 (96.4)	2204 (96.3)		
No	967 (97.4)	1070 (95.3)			82 (3.6)	85 (3.7)		
UK	169	0			0	0		
Contraception method, *n* (%)			**< 0.001**	**0.258**			**0.001**	**0.226**
No contraception	133 (13.4)	224 (19.9)			315 (13.8)	438 (19.1)		
Condom only	513 (51.7)	467 (41.6)			1152 (50.6)	968 (42.3)		
OCP only	12 (1.2)	31 (2.8)			27 (1.2)	71 (3.1)		
Condom + OCP	51 (5.1)	45 (4.0)			106 (4.7)	96 (4.2)		
Others	284 (28.6)	356 (31.7)			676 (29.7)	716 (31.3)		
UK	169	0			0	0		
History of other vaccine, *n* (%)			**0.004**	**0.124**			0.716	0.018
Yes	1043 (99.5)	1103 (98.2)			2255 (99.1)	2263 (98.9)		
No	5 (0.5)	20 (1.8)			20 (0.9)	25 (1.1)		
UK	114	0			0	0		

*Note:* The bold values mean that there is a statistical significance between two groups.

Abbreviations: OCP, oral contraceptive pill; UK, unknown.

All these characteristics demonstrated statistically significant differences between the two groups (*p* < 0.05, SMD > 0.1) (Table 1). To mitigate the potential influence of confounding factors on subsequent analysis, inverse probability treatment weighting (IPTW) was employed to balance between‐group differences. After balancing, no significant differences were observed in race, age, education level, pregnancy history, smoking history, and other vaccination history (*p* > 0.05, SMD < 0.1). However, differences remained in personal monthly income and contraceptive methods (Table 1).

### 3.2 Factors Influencing the Effectiveness of HPV Vaccine Against HPV Infection

For participants with known HPV results, infection rates of various HPV types were calculated and compared between groups. The HPV16/18 infection rate (3.0% vs. 6.8%, *p* = 0.003) and infection rate for HPV types covered by the 9‐valent vaccine (9.2% vs. 14.2%, *p* = 0.009) were significantly lower in the vaccinated group compared to the unvaccinated group in the original population. The VE of the HPV vaccine for preventing HPV16/18 infections was 57.8% (95% CI 24.3%–76.4%), and for preventing infections by 9‐valent types was 38.6% (95% CI 11.5%–57.5%).

Following IPTW application, the infection rates of HPV16/18 (3.1% vs. 6.4%, *p* < 0.001), and 9‐valent HPV types (10.1% vs. 13.5%, *p* = 0.011) and HPV31/33/45 (1.0% vs. 2.3%, *p* = 0.016) were all significantly lower in the vaccinated group compared to the unvaccinated group. The corresponding VE for preventing HPV16/18 infections was 53.9% (95% CI 30.5%–69.4%), for preventing 9‐valent types infections was 28% (95% CI 7.1%–44.3%), and for preventing HPV31/33/45 infections was 56.9% (95% CI 12.7%–78.7%) (Table 2).

**TABLE 2:** HPV infection rates and the HPV vaccine efficacy in vaccinated and unvaccinated groups.

	Before IPTW					After IPTW				
	Vaccinated *n* = 526	Unvaccinated *n* = 818	RR (95% CI)	VE (95% CI)	*p*	Vaccinated *n* = 1054	Unvaccinated *n* = 1601	RR (95% CI)	VE (95% CI)	*p*
hrHPV, *n* (%)	90 (17.1)	163 (19.9)	0.829 (0.624–1.102)	17.1% (−10.2%–37.6%)	0.197	193 (18.3)	307 (19.2)	0.945 (0.774–1.154)	5.5% (−15.4%–22.6%)	0.577
HPV16/18, *n* (%)	15 (3.0)	54 (6.8)	0.422 (0.236–0.757)	57.8% (24.3%–76.4%)	**0.003**	31 (3.1)	101 (6.4)	0.461 (0.306–0.695)	53.9% (30.5%–69.4%)	**< 0.001**
9 valent HPV, *n* (%)	45 (9.2)	111 (14.2)	0.614 (0.425–0.885)	38.6% (11.5%–57.5%)	**0.009**	98 (10.1)	206 (13.5)	0.720 (0.557–0.929)	28.0% (7.1%–44.3%)	**0.011**
HPV31/33/45, *n* (%)	5 (1.0)	19 (2.4)	0.416 (0.154–1.122)	58.4% (−12.2%–84.6%)	0.074	10 (1.0)	36 (2.3)	0.431 (0.213–0.873)	56.9% (12.7%–78.7%)	**0.016**

*Note:* When calculating the HPV infection rate, the number of individuals infected with a specific HPV type was considered relative to the total number of individuals in chance. For instance, when calculating the HPV16/18 infection rate, high‐risk types without specific typing were excluded. The bold values indicated a statistical significance between two groups.

Abbreviations: hrHPV, high‐risk HPV types; VE, vaccine effectiveness.

We conducted logistic regression analysis to explore factors influencing HPV16/18 infections. In the univariate analysis, protective factors against HPV16/18 infection included HPV vaccination (OR 0.422, 95% CI 0.236–0.757, *p* = 0.004), having a master's degree or higher (OR 0.060, 95% CI 0.009–0.407, *p* = 0.004), a monthly income of 8001–10 000 CNY (OR 0.232, 95% CI 0.077–0.693, *p* = 0.009), and a monthly income above 10 000 CNY (OR 0.188, 95% CI 0.055–0.644, *p* = 0.008). Risk factors included a higher number of pregnancies (OR 1.154, 95% CI 1.005–1.325, *p* = 0.042) and oral contraceptive use alone (OR 3.831, 95% CI 1.213–12.098, *p* = 0.022). Following IPTW, increased age (OR 0.973, 95% CI 0.948–0.998, *p* = 0.036) emerged as an additional protective factor, while smoking (OR 2.708, 95% CI 1.414–5.186, *p* = 0.003) was identified as a risk factor for HPV16/18 infection (Table 3).

**TABLE 3:** Univariate analysis: factors influencing HPV 16/18 infection.

	Before IPTW		After IPTW	
	OR (95% CI)	*p*	OR (95% CI)	*p*
Age	0.989 (0.954–1.024)	0.524	0.973 (0.948–0.998)	**0.036**
Race	0.745 (0.265–2.092)	0.577	0.737 (0.337–1.613)	0.445
Education level				
Elementary school	Reference		Reference	
Junior high school	0.444 (0.082–2.422)	0.349	0.497 (0.115–2.160)	0.351
High school	0.455 (0.092–2.260)	0.335	0.635 (0.159–2.532)	0.520
Undergraduate	0.227 (0.047–1.089)	0.064	0.318 (0.081–1.241)	0.099
Graduate or higher	0.060 (0.009–0.407)	**0.004**	0.066 (0.013–0.329)	**0.001**
Monthly income (CNY)				
None	Reference		Reference	
< 2000	0.945 (0.232–3.853)	0.937	0.741 (0.237–2.323)	0.608
2001–5000	0.579 (0.247–1.361)	0.210	0.817 (0.441–1.513)	0.520
5001–8000	0.373 (0.153–0.908)	0.030	0.581 (0.309–1.092)	0.092
8001–10 000	0.232 (0.077–0.693)	**0.009**	0.236 (0.102–0.544)	**0.001**
> 10 000	0.188 (0.055–0.644)	**0.008**	0.230 (0.096–0.547)	**0.001**
Smoking history	2.396 (0.984–5.830)	0.054	2.708 (1.414–5.186)	**0.003**
Pregnancy	1.181 (0.610–2.286)	0.622	0.949 (0.609–1.478)	0.817
Times of pregnancy	1.154 (1.005–1.325)	**0.042**	1.110 (1.004–1.228)	**0.042**
Times of birth	1.083 (0.800–1.467)	0.606	1.030 (0.826–1.285)	0.792
Contraception method				
None	Reference		Reference	
Condom only	0.712 (0.346–1.464)	0.356	0.751 (0.442–1.278)	0.292
OCP only	3.831 (1.213–12.098)	**0.022**	3.875 (1.682–8.926)	**0.001**
Condom + OCP	0.947 (0.254–3.522)	0.935	1.067 (0.423–2.688)	0.891
Others	1.180 (0.559–2.491)	0.664	1.216 (0.699–2.116)	0.489
HPV vaccine	0.422 (0.236–0.757)	**0.004**	0.463 (0.307–0.699)	**< 0.001**

*Note:* The bold values mean that there is a statistical significance between two groups.

Abbreviation: OCP, oral contraceptive pill.

The multivariate analysis incorporated factors that were statistically significant in the univariate analysis. Oral contraceptive use alone (OR 4.146, 95% CI 1.250–13.749, *p* = 0.020) and monthly income above 10 000 CNY (OR 0.221, 95% CI 0.050–0.973, *p* = 0.046) were identified as independent risk factors for HPV16/18 infection, with no other significant factors observed. After IPTW, independent protective factors against HPV16/18 infection included increased age (OR 0.958, 95% CI 0.929–0.988, *p* = 0.007), having a master's degree or higher (OR 0.099, 95% CI 0.017–0.565, *p* = 0.009), and HPV vaccination (OR 0.502, 95% CI 0.329–0.766, *p* = 0.001). Risk factors included a higher number of pregnancies (OR 1.132, 95% CI 1.001–1.280, *p* = 0.049) and oral contraceptive use alone (OR 3.501, 95% CI 1.467–8.357, *p* = 0.005). The multivariate analysis after IPTW confirmed the HPV vaccine effectiveness against HPV 16/18 (Table 4).

**TABLE 4:** Multivariate analysis: factors influencing HPV 16/18 infection.

	Before IPTW		After IPTW	
	OR (95% CI)	*p*	OR (95% CI)	*p*
Age	/	/	0.958 (0.929–0.988)	**0.007**
Education level				
Elementary school	Reference		Reference	
Junior high school	0.288 (0.045–1.836)	0.188	0.378 (0.082–1.735)	0.211
High school	0.579 (0.102–3.293)	0.538	0.569 (0.133–2.437)	0.448
Undergraduate	0.414 (0.069–2.480)	0.334	0.355 (0.081–1.550)	0.169
Graduate or higher	0.174 (0.020–1.510)	0.113	0.099 (0.017–0.565)	**0.009**
Monthly income (CNY)				
None	Reference		Reference	
< 2000	0.824 (0.191–3.546)	0.794	0.749 (0.228–2.458)	0.634
2001–5000	0.609 (0.245–1.513)	0.286	0.989 (0.519–1.885)	0.973
5001–8000	0.479 (0.178–1.294)	0.147	0.904 (0.454–1.801)	0.773
8001–10 000	0.372 (0.113–1.229)	0.105	0.438 (0.180–1.063)	0.068
> 10 000	0.221 (0.050–0.973)	**0.046**	0.504 (0.195–1.303)	0.157
Smoking history	/	/	1.818 (0.899–3.678)	0.096
Times of pregnancy	1.003 (0.838–1.200)	0.975	1.132 (1.001–1.280)	**0.049**
Contraception method				
None	Reference		Reference	
Condom only	0.776 (0.358–1.685)	0.522	0.976 (0.561–1.699)	0.933
OCP only	4.146 (1.250–13.749)	**0.020**	3.501 (1.467–8.357)	**0.005**
Condom + OCP	0.673 (0.141–3.211)	0.620	0.954 (0.369–2.466)	0.923
Others	0.948 (0.424–2.117)	0.896	1.158 (0.652–2.057)	0.616
HPV vaccine	0.637 (0.332–1.223)	0.176	0.502 (0.329–0.766)	**0.001**

*Note:* The bold values mean that there is a statistical significance between two groups.

Abbreviation: OCP, oral contraceptive pill.

### 3.3 HPV Vaccine Effectiveness Against New HPV Infections

To evaluate the protective effect of the HPV vaccine, we assessed new hr‐HPV infection incidence among 476 evaluable participants from the vaccinated group (post‐vaccination HPV positive with pre‐vaccination results or post‐vaccination HPV negative) and 818 participants with HPV test results from the unvaccinated group. A new HPV infection was defined as hr‐HPV positivity post‐vaccination in participants who were negative for the corresponding HPV types pre‐vaccination.

In the original population, new infection rates in the vaccinated group were significantly lower than those in the unvaccinated group for hr‐HPV (6.1% vs. 19.9%, *p* < 0.001), HPV16/18 (1.5% vs. 6.8%, *p* < 0.001), 9‐valent HPV types (3.3% vs. 14.2%, *p* < 0.001), and HPV31/33/45 (0.4% vs. 2.4%, *p* = 0.009). The vaccine effectiveness (VE) was 73.9% (95% CI 60.6%–82.7%) for preventing new hr‐HPV infections, 79.1% (95% CI 53.6%–90.5%) for HPV16/18, 79.5% (95% CI 64.5%–88.2%) for 9‐valent types, and 82.4% (95% CI 24.0%–95.9%) for HPV31/33/45 (Table 5).

**TABLE 5:** HPV infection rates and HPV vaccine efficacy in vaccinated and unvaccinated groups with the corresponding HPV negative before vaccination.

	Before IPTW					After IPTW				
	Vaccinated *n* = 476	Unvaccinated *n* = 818	RR (95% CI)	VE (95% CI)	*p*	Vaccinated *n* = 945	Unvaccinated *n* = 1601	RR (95% CI)	VE (95% CI)	*p*
hrHPV, *n* (%)	29 (6.1)	163 (19.9%)	0.261 (0.173–0.394)	73.9% (60.6%–82.7%)	**< 0.001**	61 (6.5)	307 (19.2)	0.291 (0.218–0.388)	70.9% (61.2%–78.2%)	**< 0.001**
HPV16/18, *n* (%)	7 (1.5%)	54 (6.8%)	0.209 (0.095–0.464)	79.1% (53.6%–90.5%)	**< 0.001**	15 (1.6)	101 (6.4)	0.239 (0.138–0.413)	76.1% (58.7%–86.2%)	**< 0.001**
9 valent HPV, *n* (%)	15 (3.3%)	111 (14.2%)	0.205 (0.118–0.355)	79.5% (64.5%–88.2%)	**< 0.001**	31 (3.4)	206 (13.5)	0.227 (0.154–0.335)	77.3% (66.5%–84.6%)	**< 0.001**
HPV31/33/45, *n* (%)	2 (0.4%)	19 (2.4%)	0.176 (0.041–0.760)	82.4% (24.0%–95.9%)	**0.009**	4 (0.4)	36 (2.3)	0.184 (0.065–0.519)	81.6% (48.1%–93.5%)	**< 0.001**

*Note:* The bold values mean that there is a statistical significance between two groups.

Abbreviations: hrHPV, high‐risk HPV types; RR, risk ratio; VE, vaccine effectiveness.

Following IPTW adjustment, results remained consistent: new infection rates were significantly lower in the vaccinated group for hr‐HPV (6.5% vs. 19.2%, *p* < 0.001), HPV16/18 (1.6% vs. 6.4%, *p* < 0.001), 9‐valent types (3.4% vs. 13.5%, *p* < 0.001), and HPV31/33/45 (0.4% vs. 2.3%, *p* < 0.001). The corresponding VE values were 70.9% (95% CI 61.2%–78.2%) for preventing new hr‐HPV infections, 76.1% (95% CI 58.7%–86.2%) for HPV16/18, 77.3% (95% CI 66.5%–84.6%) for 9‐valent types, and 81.6% (95% CI 48.1%–93.5%) for HPV31/33/45. Detailed results are presented in Table 5.

### 3.4 HPV Vaccine Effectiveness for Different Pre‐Vaccination Infection Status

To investigate the significance of pre‐vaccination HPV testing, we categorized 526 vaccinated participants (from *N* = 1162) based on their pre‐vaccination HPV testing status: no HPV testing (*n* = 155), negative for hr‐HPV (*n* = 340), and positive for hr‐HPV (*n* = 31). We then compared post‐vaccination HPV positivity rates for various HPV types across these groups. Women without pre‐vaccination HPV testing showed significantly higher post‐vaccination positivity rates for hr‐HPV (8.2% vs. 27.7%, *p* < 0.001, RR 4.278, 95% CI 2.537–7.215), and 9‐valent HPV types (4.3% vs. 13.6%, *p* < 0.001, RR 3.477, 95% CI 1.690–7.155) compared to women who were hr‐HPV‐negative pre‐vaccination. As shown in Table 6, these differences were statistically significant. Since undetected hr‐HPV infections could not be ruled out in women without pre‐vaccination HPV testing, a comparison was made of new hr‐HPV infection rates between women who were hr‐HPV negative pre‐vaccination and those who were hr‐HPV positive. The analysis revealed no significant differences in new HPV infection rates, except for HPV31/33/45 infections (0.3% vs. 3.8%, *p* = 0.021) (Table 6).

**TABLE 6:** HPV infection rates for women hrHPV negative or HPV unknown before vaccination.

	hrHPV negative pre‐vaccination	HPV unknown pre‐vaccination	hrHPV positive pre‐vaccination	HPV unknown vs. HPV negative		HPV positive vs. HPV negative	
				*p*	RR (95% CI)	*p*	RR (95% CI)
hrHPV	28 (8.2%)	43 (27.7%)	1 (3.8%)	**< 0.001**	4.278 (2.537–7.215)	0.425	0.446 (0.058–3.414)
HPV16/18	7 (2.1%)	4 (2.7%)	0 (0.0%)	0.680	1.299 (0.374–4.506)	0.455	/
9 valent HPV	14 (4.3%)	19 (13.6%)	1 (4.0%)	**< 0.001**	3.477 (1.690–7.155)	0.939	0.923 (0.116–7.318)
HPV31/33/45	1 (0.3%)	3 (2.1%)	1 (3.8%)	0.049	7.117 (0.734–69.021)	**0.021**	13.00 (0.789–214.101)

*Note:* The bold values mean that there is a statistical significance between two groups.

Abbreviations: hrHPV, high‐risk HPV types; RR, risk ratio.

## 4 Discussion

### 4.1 Factors Influencing Women's Decision to Receive HPV Vaccine

Our analysis revealed that vaccinated women were more likely to be younger, have a higher education level, a higher monthly income, fewer pregnancies and births, and more frequent condom use for contraception compared to unvaccinated women (Tables 3 and 4). These demographic and behavioral factors appear to influence HPV vaccine acceptance and uptake. Previous studies have also demonstrated a positive correlation between HPV vaccination rates and both family education and household income [31]. Given that HPV vaccination in China is primarily self‐funded, women of higher socioeconomic status may have greater financial capacity and willingness to pursue vaccination. Additionally, women who regularly use condoms for contraception may have heightened awareness of sexually transmitted infection prevention, including HPV, potentially increasing their likelihood of seeking HPV vaccination. Consistent with previous research on HPV vaccine knowledge and acceptance, our findings suggest that enhancing public awareness about HPV prevalence, associated risks, and vaccine benefits, while simultaneously improving vaccination payment models, may increase HPV vaccine uptake among Chinese women [32, 33].

### 4.2 Protective Effect of the HPV Vaccine Among Chinese Women

The efficacy of the HPV vaccination in preventing HPV incidental and persistent infections, CIN, and cervical cancer has been well‐documented through numerous studies both within China and internationally [30, 34]. While clinical trials maintain strict participant controls, our study recruited participants primarily from gynecologic outpatient clinics and physical examination centers, better reflecting real‐world conditions and providing more generalizable evidence of vaccination impact in the general population. Our data confirmed vaccine efficacy, with particularly robust protective effects observed against newly acquired post‐vaccination HPV infections (Table 2). This finding aligns with previous randomized controlled trials that demonstrated enhanced vaccine efficacy among women who were HPV‐negative for the corresponding types prior to vaccination [35].

Following inverse probability of treatment weighting (IPTW), our analysis revealed vaccine effectiveness (VE) of 76.1% (95% CI 58.7%–86.2%) against newly acquired HPV16/18 infections (Table 5), comparable to the VE of 82.0% (95% CI 62.5%–92.4%) reported in a previous clinical trial of bivalent vaccine among Asian women [35]. Furthermore, the observed VE against new hr‐HPV infections was 70.9% (95% CI 61.2%–78.2%), exceeding the previously reported VE of 20.7% (95% CI −0.4% to 37.4%) from the same clinical trial, and this enhanced effectiveness likely reflects the inclusion of women who received the 9‐valent vaccine in our study population [35, 36].

### 4.3 Factors Affecting HPV16/18 Infection

Univariate and multivariate analyses revealed that, beyond HPV vaccination, increased age and higher educational attainment—specifically at the master's degree level or above—serve as independent protective factors against HPV16/18 infection. Previous research has demonstrated that HPV infection rates among Chinese women typically exhibit two peaks: one in women under 30 and another in those over 50 years of age [25]. As our study population included women aged 18–50, encompassing only the first peak, age emerged as a protective factor. Women with advanced education typically possess greater knowledge of HPV infection, potentially reducing both their infection risk and infection duration, highlighting the importance of public education regarding HPV [37].

Furthermore, our analysis identified an increased number of pregnancies and exclusive use of oral contraceptives as independent risk factors for HPV16/18 infection. While previous research involving 7718 women has established an association between hormonal contraceptive use and HPV‐16 infection, our findings regarding oral contraceptive use should be interpreted cautiously due to the small sample size (*n* = 43) and wide confidence interval (OR 3.83, 95% CI: 1.21–12.10) [38]. Additional studies with larger cohorts are needed to validate this association. The correlation between multiple pregnancies and increased HPV infection risk likely reflects greater exposure through unprotected sexual activity [4]. Although previous research studies suggest oral contraceptives may influence early events in HPV infection natural history, oral contraceptive users tend to use condoms less frequently, despite condoms' demonstrated efficacy in reducing HPV transmission [39, 40]. Future research should incorporate detailed sexual behavior data to better delineate these relationships.

### 4.4 HPV Vaccine Efficacy by Different Pre‐Vaccination Infection Status

Most existing clinical trials related to HPV vaccines have restrictions on HPV‐DNA and serum HPV antibody status at the time of enrollment, or they performed efficacy analyses mainly among patients who were negative for the relevant HPV type at baseline [41, 42]. However, as the ACIP does not recommend routine HPV and TCT testing prior to HPV vaccination, and a positive HPV status is not an absolute contraindication for vaccination, many women are vaccinated without prior HPV testing, some of whom may be HPV positive [20]. This situation is particularly prevalent among women undergoing catch‐up vaccinations [20, 43]. Our analysis revealed that individuals without pre‐vaccination HPV testing had higher infection risk for hr‐HPV (RR 4.278, 95% CI 2.537–7.215), HPV16/18 (RR 1.299, 95% CI 0.374–4.506), and 9‐valent types (RR 3.477, 95% CI 1.690–7.155) compared to those who tested hr‐HPV negative pre‐vaccination (Table 6). However, the possibility of undetected pre‐existing HPV infection persisting until post‐vaccination detection cannot be excluded.

To address this uncertainty, an analysis was conducted of post‐vaccination HPV results in women who were HPV‐positive pre‐vaccination. When considering only new infections, results showed no significant difference in vaccine protective effect between pre‐vaccination hr‐HPV‐positive and hr‐HPV‐negative women, with no statistically significant difference in post‐vaccination new HPV infection rates. There is no clear consensus on the preventive effect of the HPV vaccine on reinfection after the clearance of a pre‐existing HPV infection, and the HPV vaccine is a prophylactic vaccine without a clear effect on the clearance of HPV [22, 44]. Previous studies have shown that the protective effect of the HPV vaccine against CIN2+ lesions is less pronounced in populations without restrictions on pre‐vaccination HPV infection status compared to those confirmed HPV16/18‐negative before vaccination [30]. Thus, vaccination with the HPV vaccine in women with unknown or positive HPV results pre‐vaccination can still provide prevention against other HPV types not yet infected. As the HPV vaccine is prophylactic and does not demonstrably aid in the clearance of existing HPV infections, the overall benefits of vaccination in such cases may be comparatively constrained. Therefore, regular monitoring is still required even after HPV vaccination, giving pre‐vaccination HPV testing certain clinical significance.

Recent studies have demonstrated that post‐conization vaccination significantly reduces CIN2+ recurrence and subsequent HPV infections and related cervical lesions after excisional treatment [45, 46, 47, 48]. Additionally, studies have indicated that women who underwent hysterectomy for CIN2+ or early‐stage cervical cancer continue to benefit from HPV vaccination's protective effects against lower genital tract dysplasia [49]. These findings, combined with the observation of reliable vaccine protective efficacy against previously unencountered HPV types, support the integration of vaccination into comprehensive post‐treatment management protocols.

### 4.5 Strength and Limitations

Our study provides valuable insights into the relationship between socioeconomic factors, HPV knowledge, and vaccination willingness, offering strategic guidance for improving HPV vaccine coverage in China. By recruiting participants from hospitals across different tiers and including women with different pre‐vaccination HPV infection statuses, our research closely reflects real‐world conditions. This diversity enables gynecologists to more effectively evaluate vaccine effectiveness in the general population. The study elucidates HPV vaccine protective effects across a broad demographic spectrum, including women of various ages and HPV infection histories, contributing to a comprehensive understanding of vaccine efficacy. Additionally, our analysis of factors influencing HPV16/18 infection among vaccinated women provides evidence‐based recommendations for risk reduction and HPV education strategies.

Our study still has some limitations. First, as data were collected from three medical institutions within a single administrative region, our findings may not fully represent the broader Chinese female population. The conclusions are exploratory and require validation across diverse geographical regions. Second, while questionnaire‐based data collection of HPV and TCT results was supplemented by hospital electronic database verification, many participants had HPV results without specific typing, introducing analytical complexities. Finally, the relatively modest sample size and absence of long‐term follow‐up limit our ability to draw definitive conclusions about vaccine protective effects in pre‐vaccination HPV positive populations. Given these limitations, our findings should be considered preliminary. Future research will include expanding the sample size, broadening geographical representation, and extending follow‐up periods to generate more robust evidence regarding HPV vaccine efficacy across different contexts and its broader public health impact.

## 5 Conclusion

In conclusion, our study demonstrates that improvements in socioeconomic conditions and heightened awareness of HPV risks and prevalence could significantly enhance HPV vaccine uptake. Additionally, we identified age, parity, exclusive use of oral contraceptives, and higher educational attainment (master's degree or higher) as independent predictors of HPV16/18 infection, alongside HPV vaccination status. Furthermore, our analysis suggests that pre‐vaccination HPV infection status does not directly affect the vaccine's protective efficacy against types not previously encountered.

## Author Contributions

**Lucia Li:** investigation, formal analysis, writing – original draft; **Haiyue Wu:** investigation, writing – original draft; **Minghui Qiu, Yingzhen Liu, Yufei Shen, Yingnan Lu, Xinxin Wen, Siyu Yang, Kehan Zou, Hui Zhang, Yangzong Gesang, and Haojie Huang:** investigation; **Yibo Chen, Zhanjun Shen, Kun Fu, Chao Zhao and Pengming Sun:** conceptualization, investigation; **Lisha Wu:** conceptualization, writing – review and editing; **Yu Zhang:** conceptualization, writing – review and editing.

## Ethics Statement

This study and all its methods will be carried out in accordance with the ethical standards of the institution and the National Commission for Human Experimentation, as well as the 1964 Declaration of Helsinki and its subsequent amendments or equivalent. The study was approved by the ethics committee of Xiangya Hospital, Central South University, China (ethics number 202103233). This committee is independent and not related to any affiliation of the authors.

## Consent

The study will be explained to the patients by the gynecologist, and an informed consent form will be obtained from all participants. All authors of this research paper have directly participated in the planning, execution, or analysis of the study. All authors of this paper have read and approved the final version submitted.

## Conflicts of Interest

The authors declare no conflicts of interest.

## Supporting information


**Table S1:** Content of the questionnaire.

## Data Availability

The data that support the findings of this study are available on request from the corresponding author. The data are not publicly available due to privacy or ethical restrictions.
