# Supplementary material for: Influence of Pre‐Vaccination HPV Status on Vaccine Effectiveness Among Chinese Women: A Multicenter Cross‐Sectional Study
Source: Cancer Rep (Hoboken). 2025 Aug 28;8(9):e70294. doi: 10.1002/cnr2.70294 (PMC12394005; doi:10.1002/cnr2.70294)
Supplement: Supplementary file 1 — Table S1: Content of the questionnaire. [file CNR2-8-e70294-s001.docx]

**Table S1 content of the questionnaire**

| Category | Number of Questions | Target Group | Questions Included |
| --- | --- | --- | --- |
| Basic Information | 2 | All participants | Medical institution, ID number |
| Demographic Characteristics | 13 | All participants | Race, age, marital status, pregnancy history, number of pregnancies, number of childbirths, place of residence, household registration, occupation, highest education level, average monthly income, contraceptive method, smoking history |
| HPV Vaccination | 6 | Vaccinated participants | HPV vaccine type, date of first dose, main reason for vaccination, sexual history before vaccination, adverse reactions, completion of vaccination schedule |
| HPV/TCT Testing | 6 | Vaccinated participants | HPV test within 12 months before vaccination, HPV test result, date of pre-vaccination HPV test, TCT test within 12 months before vaccination, TCT test result, date of pre-vaccination TCT test |
| HPV/TCT Testing | 6 | Participants who completed vaccination | HPV test after vaccination, HPV test result, date of post-vaccination HPV test, TCT test after vaccination, TCT test result, date of post-vaccination TCT test |
| HPV/TCT Testing | 6 | Unvaccinated participants | HPV test history, HPV test result, date of HPV test, TCT test history, TCT test result, date of TCT test |
